# Causal effects between circulating immune cells and heart failure: evidence from a bidirectional Mendelian randomization study

**DOI:** 10.1186/s12920-024-01827-5

**Published:** 2024-02-26

**Authors:** Rutao Bian, Xuegong Xu, Zishuang Li

**Affiliations:** 1 Zhengzhou Hospital of Traditional Chinese Medicine, Zhengzhou, China; 2 Guangzhou University of Traditional Chinese Medicine - Zhengzhou Hospital of Traditional Chinese Medicine Joint Laboratory of formulas-syndromes Research, Zhengzhou, China; 3 Henan Key Laboratory of Traditional Chinese Medicine Cardiovascular Disease, Zhengzhou, China

**Keywords:** Heart failure, Immune cell, Inflammation, Mendelian randomization, Lymphocytes

## Abstract

**Background:**

Heart failure (HF) is a prevalent cardiac condition characterized by high mortality and morbidity rates. Immune cells play a pivotal role as crucial biomarkers in assessing the overall immune status of individuals. However, the causal relationship between circulating immune cells and the pathogenesis of HF remains an area requiring further investigation.

**Objectives:**

The aim of this study was to investigate the genetic interactions between circulating immune cells and HF, and to further elucidate the genetic associations between different lymphocyte subsets and HF.

**Methods:**

We obtained genetic variants associated with circulating immune cells as instrumental variables (IVs) from the Blood Cell Consortium and publicly available HF summary data. We conducted additional subsets analyses on lymphocyte counts. Our study utilized two-sample and multivariate Mendelian randomization (MVMR) analysis to investigate the causal effect of immune cells on HF. The primary analysis employed inverse variance weighting (IVW) and was complemented by a series of sensitivity analyses.

**Results:**

The findings of the study showed that the IVW model demonstrated a significant correlation between an elevation in lymphocyte count and a decreased risk of HF (OR = 0.97, 95% CI, 0.94 - 1.00, *P* = 0.032). However, no such correlation was evident in the MVMR analysis for lymphocytes and HF. Furthermore, the examination of the lymphocyte subsets indicated that an increase in CD39^+^ CD4^+^ T-cell counts was notably linked to a reduced risk of HF (OR = 0.96, 95% CI, 0.95 - 0.98, *P* = 0.0002). The MVMR results confirmed that the association between CD39^+^ CD4^+^ T-cell counts and HF remained significant. There was no substantial evidence of reverse causality observed between circulating immune cells and HF.

**Conclusion:**

Our MR research provided evidence for a causal relationship between lymphocyte cell and HF. Subsets analyses revealed a causal relationship between CD39^+^ CD4^+^ T lymphocytes and HF. These findings will facilitate a future understanding of the mechanisms underlying HF.

**Supplementary Information:**

The online version contains supplementary material available at 10.1186/s12920-024-01827-5.

## Introduction

Heart failure (HF) is a complex clinical syndrome whose symptoms and signs are caused by either structural or functional abnormality of ventricular filling or ejection, with a 5-year survival rate of less than 50% and affects over 64 million people worldwide [1]. The prevalence of HF will continue to increase as the population ages, resulting in a significant economic burden on the public health system [2]. Research into and identification of modifiable risk factors is essential for the management and prevention of HF.

There has been longstanding interest in the role of inflammation in the pathophysiology of HF. Plasma levels of pro-inflammatory factors have been found to correlate with disease severity and predict poor survival in patients with chronic HF [3]. Although the exact involvement of leukocytes in the development of HF is not fully understood, it is widely accepted that leukocytes may contribute to HF by either directly coupling with cardiomyocytes or indirectly through the production of cytokines and antibodies. The immune system plays a key role in initiating an inflammatory response in ventricular remodeling, which is cardioprotective for a short period of time in the early stages of the disease, but if sustained may lead to a chronic low-grade inflammatory state that may accelerate disease progression [4]. Lymphocyte-mediated immune responses, as vital effector factors of the immune system, play a pivotal role in the progression of cardiovascular diseases [5]. Research has indicated that patients, regardless of their ejection fraction, were found to have an 82% higher mortality rate associated with lymphopenia [6]. CD39, an ectonucleotidase expressed on the surface of immune cells, primarily mediates the breakdown metabolism of cellular ATP. CD4^+^ lymphocytes are integral effector cells within the adaptive immune system. Notably, the presence of CD39^+^ CD4^+^ T cells is commonly associated with auxiliary Th17 effector function [7]. CD4^+^ T lymphocytes can exert a “double-edged sword” impact on the onset and progression of HF by secreting diverse cytokines, necessitating further exploration.

Cell counts are the most critical parameter of circulating immune cell homeostasis. Furthermore, observational studies have shown that circulating immune cells of several immune cells deviate from normal levels in patients with HF, indicating a possible relationship between immune cells and HF. However, the above findings have been summarized from phenotype-level results from observational studies and are susceptible to confounding and reverse causality. Mendelian randomization (MR) is an approach that uses genetic variation as an instrumental variable to assess causal effects between non-genetic and modifiable risk factors and disease [8, 9]. In the absence of randomized controlled trials, MR studies provide an alternative strategy for making causal inferences. This technique has been effectively utilized in various causal studies focusing on behavioral exposures and cardiovascular disease. However, there are no published MR studies on this topic.

In this study, a two-sample MR approach was employed to genetically validate the interaction between immune cells and HF. Single nucleotide polymorphisms (SNPs) data from a large genome-wide association study (GWAS) of haematological traits were selected as instrumental variables (IVs) for exposure, allowing us to investigate the association between different lymphocyte subsets and HF. The primary objective of this study is to enhance our understanding of the inflammatory mechanisms involved in the onset and development of HF and to explore the causal relationships underlying these mechanisms.

## Methods

### Study design

This study comprises three parts [10]. Firstly, a two-sample MR was utilized to estimate whether total peripheral leukocytes contribute to the development of HF. Secondly, we examined the causal relationship between identified lymphocyte subsets and HF. Finally, we applied the same method to investigate whether HF affects the counts of these leukocytes.

### Data sources

A two-sample MR study was conducted to systematically determine the causal effects of different peripheral blood immune cell or lymphocyte subsets on the risk of HF. For the exposure phenotypes were obtained from the IEU Open GWAS project (https://gwas.mrcieu.ac.uk/).The pooled statistics were obtained from a recent large-scale GWAS of blood cell traits by the Blood Cell Consortium (BCX), which included 563,085 participants of European ancestry [11]. The GWAS provided genetic variants associated with circulating white blood cell, lymphocyte, monocyte, neutrophil, eosinophil, and basophil counts (Supplementary Table 1).

The identification of lymphocyte subsets, including CD4, CD8, NK cells, and B cells, has been extensively employed in the immune assessment, diagnosis, and treatment of infectious diseases, progressing into a multidisciplinary approach for immune monitoring. This method can determine the severity of HF and function as an indicator for monitoring treatment effects. We conducted searches for relevant GWAS datasets, ultimately incorporating HLA DR^+^ Natural Killer cells, Resting CD4 regulatory T cells, CD8^+^ Natural Killer T cells, Secreting CD4 regulatory T cells, Natural Killer T cells, and CD39^+^ CD4^+^ T cells, B cells, Memory B cells, Naive-mature B cells, and IgD^+^ CD38^+^ B cells for this study [12]. The keyword “heart failure” was utilized to search the GWAS catalog database (https://www.ebi.ac.uk/gwas/). Data from participants of European descent were selected, specifically opting for the dataset with the largest sample size. In our study of GWAS data on HF, we conducted a meta-analysis based on the research published by Levin MG et al. [13]. The cases and controls were sourced from six independent cohorts or consortia (HERMES, eMERGE, Mount Sinai BioMe, FinnGen, Penn Medicine Biobank, and Geisinger DiscovEHR), encompassing a total of 115,150 cases and 1,550,331 controls. To minimize population stratification bias, only GWAS datasets involving individuals of European ancestry were included in this study.

### Genetic instruments selection criteria for HF

We instituted a stringent sequential screening procedure to identify SNPs closely associated with the target exposure and adhere to MR assumptions. The selected IV SNPs must surpass a genome-wide significance threshold of (< 5E-8). To ensure that the IVs are independent and unaffected by confounding factors, the linkage disequilibrium (LD) was assessed with a linkage disequilibrium parameter (R2) <0.001, using a clustering window of 10,000 kb and pop ="1000G EUR” to reduce the impact of strong linkage disequilibrium [14]. IVs with an F-statistic value >10 were deemed effective instrumental variables, thereby excluding the introduction of bias from weak instrumental variables, and were retained for subsequent analysis. The selection process for the HF IVs followed a similar approach. The association of SNPs with potential confounders was assessed using previous GWAS statistical summaries on gender and age in the a human genotype-phenotype association database PhenoScanner database with settings of *P* < 5E-8, R2 > 0.8(http://www.phenoscanner.medschl.cam.ac.uk/) [15]. Finally, exposure and outcome SNPs were matched for further analysis to establish genetic associations.

### Mendelian randomization analysis

MR studies use the Inverse Variance Weighted (IVW) model as the main criterion [16], supplemented by three other methods: MR-Egger regression [17], weighted median [18], and weighted mode [19], to assess the potential causal relationship between peripheral immune cells and HF in different scenarios. If the number of SNPs was less than 2, the Wald ratio was employed instead of IVW [20]. To measure the reliability and stability of the model inferences, sensitivity analyses were performed, and Cochran's Q test (*p* < 0.05) was applied to estimate the heterogeneity of the IVW model. When heterogeneity was detected among the studies, MR analyses were performed using the random effects IVW method. The MR-Egger [21] intercept test (*p* < 0.05) was used to identify outliers using the MR-PRESSO method [22]. Outliers were subsequently removed, and the MR analysis was repeated to exclude these outliers. The leave-one-out test was used for this analysis. Results were reported as odds ratios (ORs) with corresponding 95% confidence intervals (95% CIs) and *p*-values. Heterogeneity was assessed visually using scatter plots and funnel plots. R 4.0.3 software with the “TwoSampleMR” [23], the “MR-PRESSO” packages [24] and the “meta” package was used for data processing and result presentation. For Multivariate Mendelian randomization (MVMR) analysis, we used the IVW method to estimate causality.

## Results

### Effect of circulating immune cells on HF

**Fig. 1:**
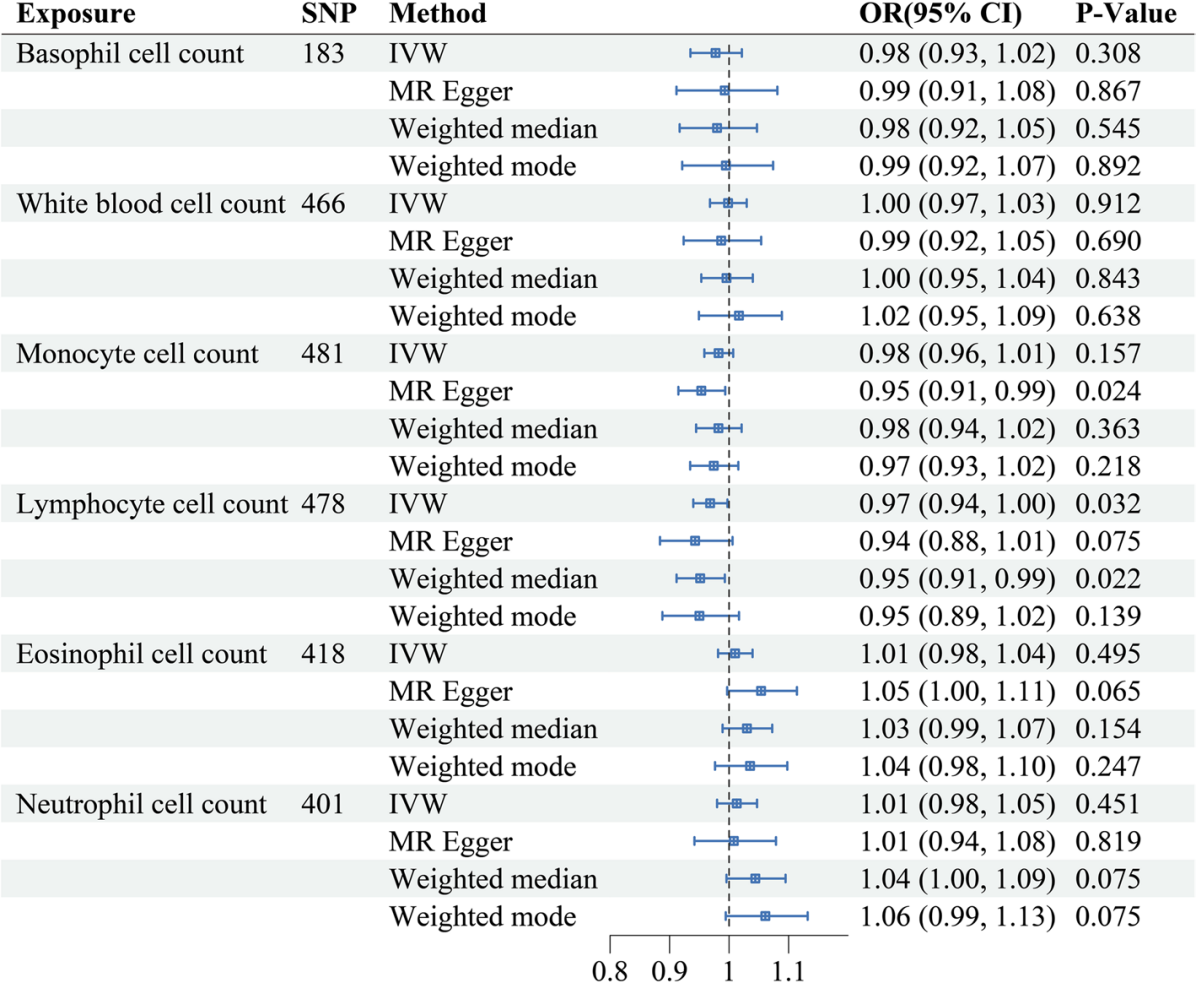
MR estimates for the causal effect of circulating immune cells and risk of HF. OR, odds ratio; CI, confidence interval

After SNP screening and matching, the initial screening identified 186 SNPs in basophils, 467 SNPs in white blood cell count, 483 SNPs in monocytes, 480 SNPs in lymphocytes, 420 SNPs in eosinophils, and 402 SNPs in neutrophils. The MR-PRESSO test identified 3 outliers in basophil count (rs1598207, rs56179563, rs56406125), 1 outlier in white blood cell count (rs11198788), 2 outliers in monocyte count (rs11235689, rs58814158), 2 outliers in lymphocyte count (rs10748526, rs11160706), 2 outliers in eosinophil count (rs4722171, rs73963711), and 1 outlier in neutrophil count (rs1412445). On further MR analysis after outlier removal, the F-statistics of all SNPs exceeded 10, indicating their suitability as powerful tools (Supplementary Tables 2– 7). A summary of the MR causal effect estimates of circulating immune cells on HF (Fig. 1). The results showed that an increase in lymphocyte count was associated with a reduced risk of HF using the IVW analysis (OR, 0.97; 95% CI, 0.94-1.00; *P* = 0.032), suggesting a protective role for lymphocytes in HF. A similar positive trend was observed using a weighted median approach (OR = 0.95, 95% CI = 0.91-0.99, *P* = 0.022). However, the results of MVMR analysis showed that this association disappeared after other risk factors were adjusted (Table 1). The limited evidence suggests no significant associations were observed between white blood cell counts, monocyte cell count, neutrophil cell count, eosinophil cell count, or basophil cell count and susceptibility to HF.

**Table 1:** MVMR estimates circulating immune cells on HF

Exposure	nSNPs	Beta	OR(95% CI)	*P*-Value
Basophil cell count	519	-0.026	0.974 (0.902, 1.051)	0.497
White blood cell count	519	0.120	1.127 (0.563, 2.260)	0.735
Monocyte cell count	519	-0.035	0.965 (0.897, 1.039)	0.343
Lymphocyte cell count	519	-0.069	0.934 (0.726, 1.201)	0.592
Eosinophil cell count	519	0.032	1.033 (0.967, 1.103)	0.338
Neutrophil cell count	519	-0.053	0.949 (0.544, 1.654)	0.852

### Sensitivity analysis of the effect of circulating immune cells on HF

**Fig. 2:**
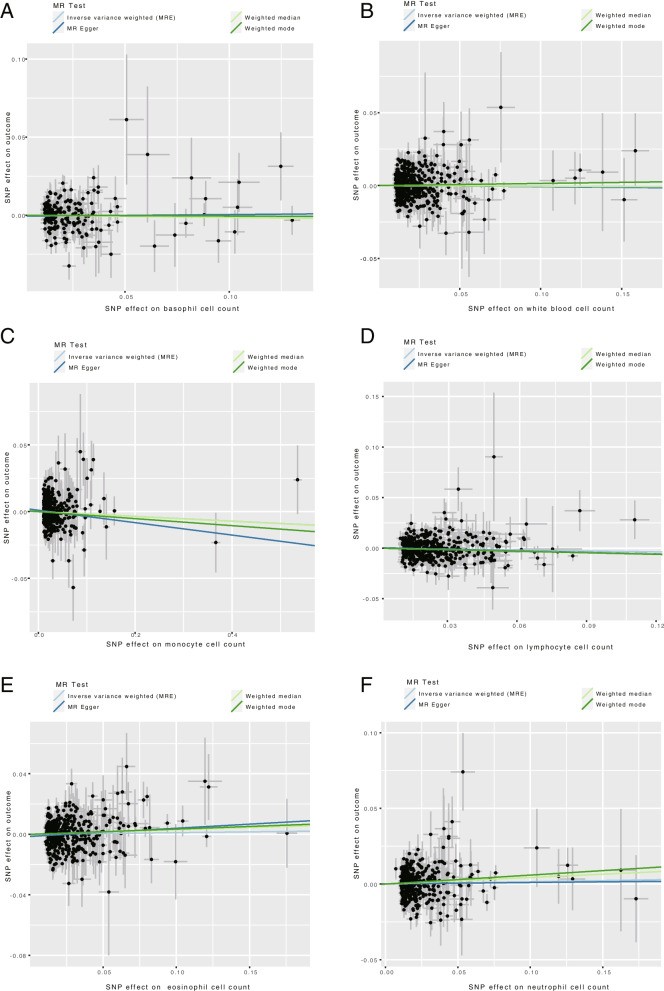
Scatter plots of the SNP effects on the circulating immune cells and HF risk. (**A**), basophil cell count; (**B**), white blood cell count; (**C**), monocyte cell count; (**D**), lymphocyte cell count; (**E**), eosinophil cell count; (**F**), neutrophil cell count

The heterogeneity and pleiotropy levels of the different omitted factors were assessed using MR-Egger and IVW regression (Supplementary Tables 8). Despite significant heterogeneity among all omitted factors (*P* < 0.05), a random effects model was utilized to estimate the MR effect size. The MR-Egger intercept results indicated that the *P *< 0.05, suggesting no significant pleiotropy was detected (Fig. 2). The leave-one-out test results revealed that excluding each of the SNPs in turn, the IVW analyses of the remaining SNPs yielded similar results to those with all SNPs included, thus indicating no SNPs had a substantial impact on the causal association estimates (Supplementary Figure 1). This further confirms the stability of the results. The six omitted factors successfully passed the MR Steiger test, which implies that the instrumental variables did not exhibit reverse causality.

### Causal effect of lymphocyte subsets on HF

**Fig. 3:**
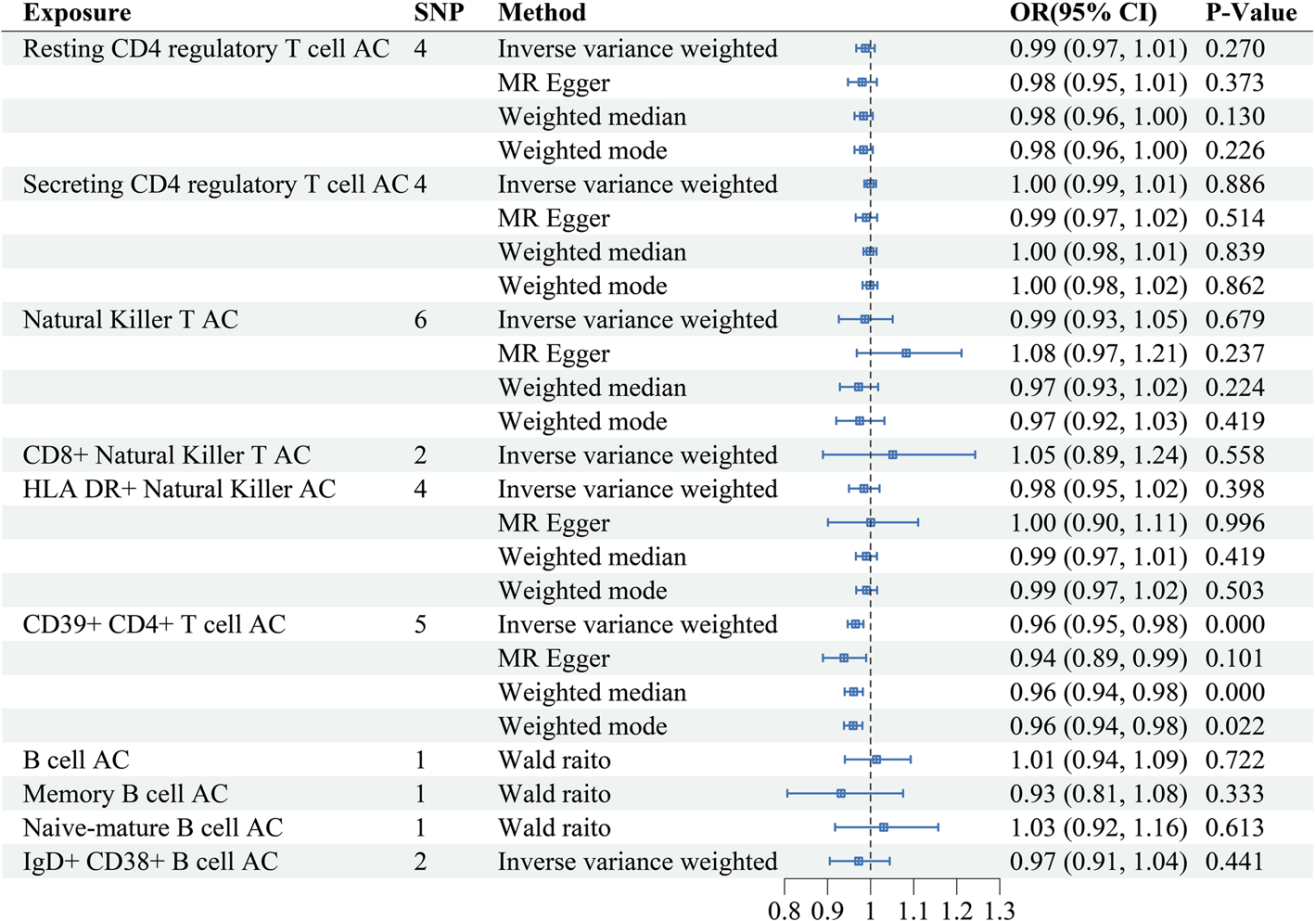
MR estimates for the causal effect of lymphocyte subsets and risk of HF. OR, odds ratio; CI, confidence interval; AC, absolute count

To assess the causal relationship between different lymphocyte subsets and HF risk, further MR analyses were performed on absolute counts of different lymphocyte subsets, including HLA-DR^+^ natural killer absolute count (AC), resting CD4 regulatory T-cell AC, CD8^+^ natural killer T-cell AC, secreting CD4 regulatory T-cell count, CD8^+^ T-cell AC, and CD39^+^ CD4^+^ T-cell AC, B cell AC, Memory B cells AC, Naive-mature B cells AC, and IgD^+^ CD38^+^ B cells AC. The F-statistics of all SNPs were greater than 10, indicating their suitability as powerful tools. The results showed that using IVW analysis, an increase in CD39^+^ CD4^+^ T-cell count was associated with a decreased risk of HF (OR = 0.96, 95% CI = 0.95-0.98, *p* = 0.0002), weighted median (OR = 0.96, 95% CI = 0.94-0.98, *p* = 0.0003), and weighted mode (OR = 0.96, 95% CI = 0.94-0.98, *p* = 0.022) showed similar positive trends (Fig. 3, Supplementary Tables 9). The MVMR analysis results show a causal relationship between an increase in CD39^+^ CD4^+^ T cell AC and a decreased risk of HF (Table 2). In addition, IVW did not provide sufficient evidence that other lymphocyte subtypes counts could influence susceptibility to HF.

**Table 2:** MVMR estimates lymphocyte subsets on HF

Exposure	nSNPs	Beta	OR(95% CI)	*P*-Value
HLA DR^+^ Natural Killer AC	27	-0.026	0.974 (0941, 1.008)	0.135
Resting CD4 regulatory T cell AC	27	0.005	1.005 (0.957, 1.055)	0.845
CD8^+^ Natural Killer T AC	27	0.091	1.096 (0.952, 1.261)	0.204
Secreting CD4 regulatory T cell AC	27	0.013	1.013 (0.969, 1.059)	0.572
Natural Killer T AC	27	-0.075	0.928 (0.828, 1.039)	0.193
CD39^+^ CD4+ T cell AC	27	-0.038	0.963 (0.936, 0.991)	0.009
B cell AC	27	0.079	1.082 (0.860, 1.362)	0.500
Memory B cell AC	27	-0.083	0.920 (0.798, 1.062)	0.255
Naive-mature B cell AC	27	0.078	1.081 (0.852, 1.373)	0.520
IgD^+^ CD38^+^ B cell AC	27	-0.093	0.911 (0.732, 1.134)	0.404

^a^*AC* Absolute count

### Sensitivity analysis of effect of lymphocyte subsets on HF

The SNP heterogeneity of the lymphocyte subsets was satisfactory using the Cochran Q test, and the MR-Egger's intercept results showed that the intercept term was not significantly different from zero, indicating that no significant pleiotropy was detected (Supplementary Figure 2). The leave-one-out test results showed that when each SNP was sequentially excluded, the IVW analyses of the remaining SNPs were similar to the analyses including all SNPs, and no SNPs were found to have a large effect on the causal association estimates, further confirming the stability of the results. The MR Steiger test indicated that there was no reverse causality in the instrumental variables.

### Effect of HF on risk of circulating immune cells

**Fig. 4:**
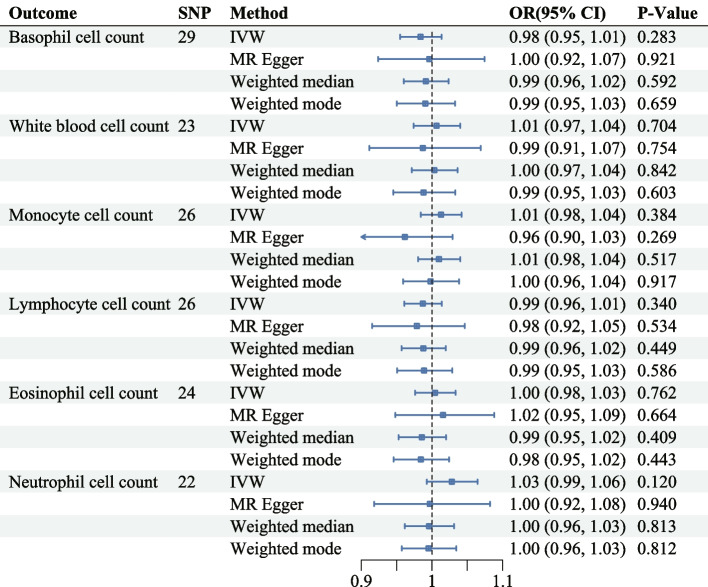
MR estimates for the causal effect of HF and risk of circulating immune cells. OR, odds ratio; CI, confidence interval; AC, absolute count

We performed a reverse analysis to assess further the causal relationship between HF and circulating immune cells. HF was considered as the exposure variable, while circulating immune cells were regarded as the dependent variable. Every IV that was selected yielded an F-statistic value higher than 10, indicating the robustness of the selected SNPs. Nevertheless, the limited evidence indicates that there is likely not a causal relationship between HF and the risk associated with peripheral immune cells (Fig. 4).

## Discussion

It is worth noting that recent studies have emphasized the significant impact of the immune system on the incidence, progression, and risk of HF [25]. However, these observational studies only confirm the involvement of immune cells and inflammation in the development of HF but fail to provide reliable evidence of a causal relationship. A mounting corpus of epidemiological and observational evidence suggests an association between peripheral immune cell count and HF risk. To elucidate this relationship, we performed a two-sample MR analysis to assess the causal impact of six peripheral WBC counts and ten lymphocyte subsets on HF. In the present study, we analyzed the causal relationship between different leukocyte counts and HF and found that an increase in lymphocyte count was associated with a reduced risk of HF (OR, 0.97; 95% CI, 0.94-1.00; P = 0.032), suggesting a protective role for lymphocytes in HF. However, an increase in white blood cell, neutrophil, eosinophil, granulocyte, or basophil counts, and HF susceptibility were not found to be significantly causally related, and the inverse MR test did not show a causal relationship. Further analysis of lymphocyte subsets showed an association between increased CD39^+^ CD4^+^ T-cell counts and reduced risk of HF (OR = 0.96, 95%CI = 0.95-0.98, *P* = 0.0002). Consistent with previous reports, we provide evidence of negative genetic causality between lymphocyte counts and HF, suggesting that lymphocytes may be protective against HF.

Neutrophils are essential components of the innate immune system and contribute to the acute-phase inflammatory response. Conversely, lymphocytes are part of the adaptive immune system and are involved in the development of autoimmune inflammation, particularly in chronic inflammatory responses [26]. HF is known to be characterized by persistent chronic inflammation, wherein T lymphocytes are believed to play a crucial role. Numerous studies have demonstrated the potential of the neutrophil-lymphocyte ratio as a prognostic predictor in HF patients [27, 28]. T cells are highly diverse, and mature T cells are classified as CD4^+^ T cells or CD8^+^ T cells depending on the presence of CD4 or CD8 surface proteins. Upon stimulation by antigens in peripheral immune organs, both CD4^+^ and CD8^+^ T cells differentiate into various subsets of effector T cells, each with distinct functions. It has been shown that CD4^+^T and CD8^+^ T cell counts are elevated in patients with chronic HF and have been shown to play an essential role in cardiac remodeling [29, 30]. Peripheral blood T lymphocyte levels can reflect the immune function and overall health status of patients. Research has indicated that in the elderly, peripheral blood lymphocyte counts are approximately 70% of those observed in younger individuals, accompanied by reduced immunoreactivity and proliferation of these cells [31].

CD39 has a crucial role in the immune system, particularly in the effector function of Th17 cells, which are known for promoting chronic inflammation and matrix degradation. The collaboration between CD39 and CD73 leads to the conversion of ATP to ADP and cAMP, ultimately generating adenosine. Adenosine interacts with various receptors (A1, A2A, A2B, and A3), initiating diverse immune responses. It stimulates immune responses via A1 and A3 receptors while exhibiting immune-suppressive effects when interacting with A2A and A2B receptors [32, 33]. Furthermore, the function of CD39^+^ CD4^+^ T cells has been studied in various diseases. Reduced frequencies of CD39^+^ T cells are linked to inflammatory diseases such as multiple sclerosis, while increased frequencies are observed in human colorectal cancer, promoting cell proliferation [34, 35]. Research has shown that CD39-activated regulatory T cells can alleviate myocardial ischemia/reperfusion injury [36]. However, the relationship between CD39^+^ CD4^+^ T cells and HF has not been sufficiently explored in previous studies, and this study establishes a causal relationship for the first time. Immune cells are believed to play a pivotal role in the pathogenesis of various forms of HF, and targeted therapies focusing on specific lymphocyte subsets may hold potential for therapeutic immunomodulation. However, more comprehensive data from randomized clinical trials are needed to support our hypothesis.

The present study is based on two-sample MR analyses of published results from a large GWAS cohort, comprising approximately 1.5 million individuals. This large sample size contributes to high statistical power. The conclusions of this study are based on genetic instrumental variables and causal inference using multiple MR analysis methods. Sensitivity testing was conducted to assess the robustness of the results and minimize potential bias from pleiotropic effects. The analyses also aimed to demonstrate the absence of confounding by horizontal pleiotropy and other factors. However, several limitations should be considered in this study. First, it should be noted that even after multiple sensitivity analyses, the assessment of horizontal pleiotropy remains limited. Second, the lack of available personal information prevented further population stratification. Third, it is important to acknowledge that the study was based on a European database, thus limiting the generalizability of the results to other ethnic groups. Finally, it is worth noting that the adoption of more relaxed thresholds for result assessment may have increased the likelihood of false positives. Additionally, prioritizing the exploration of stronger associations between immune profiles and HF should be a future research endeavor.

In conclusion, this study provides evidence indicating the potential significance of circulating lymphocyte counts in the genetic development of HF. The analysis demonstrates that specific lymphocyte subsets, such as CD39^+^ CD4^+^ T-cell counts, can serve as predictors of HF risk. These findings warrant further investigation at both the basic and clinical levels to explore in greater detail the role of specific immune cell types and genetic predispositions as biomarkers for HF risk. Such research could facilitate early diagnosis and enhance the effectiveness of treatment options for HF.

### Supplementary Information

**Supplementary material 1.** 

**Supplementary material 2.** 

## Data Availability

The datasets analysed during the current study are available in the GWAS catalog database (https://www.ebi.ac.uk/gwas/). and the IEU Open GWAS repository(https://gwas.mrcieu.ac.uk/).

## Abbreviations

HF: Heart failure
BCX: Blood Cell Consortium
GWAS: Genome-wide association studies
IVs: Instrumental variables
OR: Odds ratio
CI: Confidence interval
IVW: Inverse variance weighted
MR analysis: Mendelian randomization analysis
MVMR: Multivariate Mendelian randomization
SNPs: Single-nucleotide polymorphisms
LD: Linkage disequilibrium
MR-PRESSO: MR Pleiotropy Residual Sum and Outlier
NA: Not available
AC: Absolute count
