# Supplementary material for: Causal effects between circulating immune cells and heart failure: evidence from a bidirectional Mendelian randomization study
Source: BMC Med Genomics. 2024 Feb 26;17:62. doi: 10.1186/s12920-024-01827-5 (PMC10895739; doi:10.1186/s12920-024-01827-5)
Supplement: Supplementary file 1 — Supplementary material 1. [file 12920_2024_1827_MOESM1_ESM.docx]

**Supplementary Figure**

**Supplementary Figure 1.** **Leave-one-out plot for Lymphocyte cell count and HF.**

**Supplementary Figure 2.** **Leave-one-out plot for CD39+ CD4+ T cell absolute count and HF.**

**
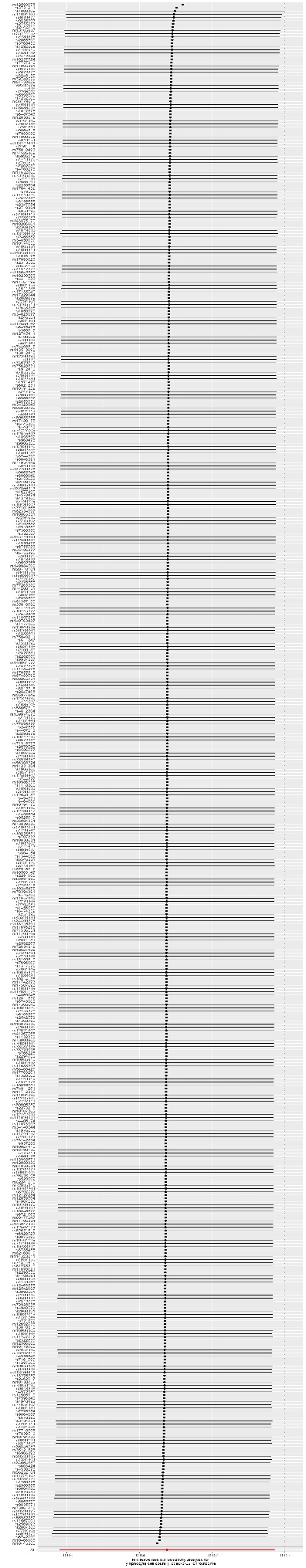
Supplementary Figure 1.** **Leave-one-out plot for Lymphocyte cell count and HF.**

**
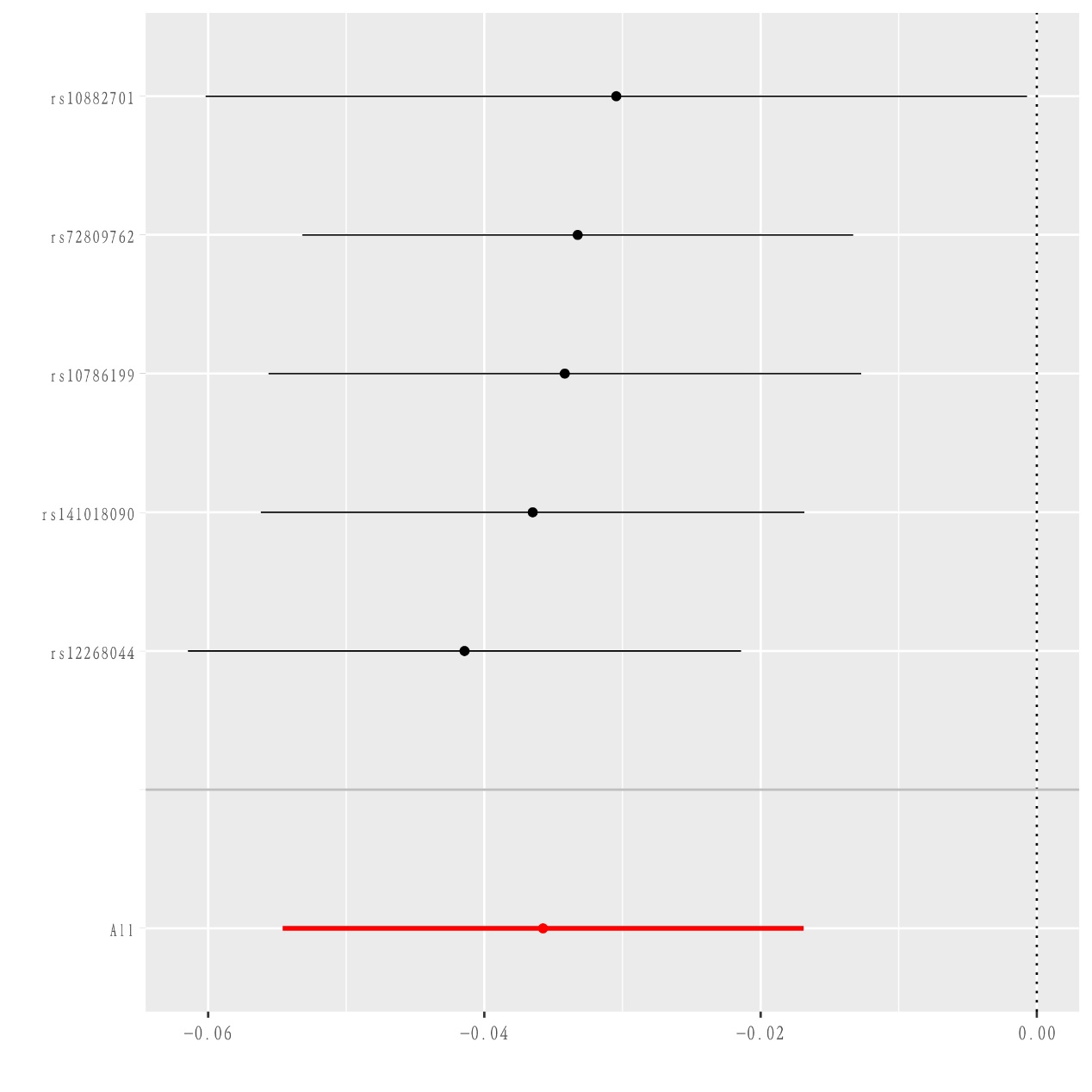
Supplementary Figure 2.** **Leave-one-out plot for CD39+ CD4+ T cell absolute count and HF.**
